# Built environment color modulates autonomic and EEG indices of emotional response

**DOI:** 10.1111/psyp.14121

**Published:** 2022-06-20

**Authors:** Isabella S. Bower, Gillian M. Clark, Richard Tucker, Aron T. Hill, Jarrad A. G. Lum, Michael A. Mortimer, Peter G. Enticott

**Affiliations:** ^1^ Cognitive Neuroscience Unit, School of Psychology, Faculty of Health Deakin University Geelong Victoria Australia; ^2^ School of Architecture and Built Environment, Faculty of Science, Engineering and Built Environment Deakin University Geelong Victoria Australia; ^3^ CADET Virtual Reality Training and Simulation Research Lab, School of Engineering, Faculty of Science, Engineering and Built Environment Deakin University Geelong Victoria Australia

**Keywords:** cave automatic virtual environment, electroencephalography, environmental psychology, frequency analyses, heart rate variability, interior built environment design, respiration, skin conductance, virtual reality, visual perception

## Abstract

Understanding built environment exposure as a component of environmental enrichment has significant implications for mental health, but little is known about the effects design characteristics have on our emotions and associated neurophysiology. Using a Cave Automatic Virtual Environment while monitoring indoor environmental quality (IEQ), 18 participants were exposed to a resting state (black), and two room scenes, control (white) and condition (blue), to understand if the color of the virtual walls affected self‐report, autonomic nervous system, and central nervous system correlates of emotion. Our findings showed that exposure to the chromatic color condition (blue) compared to the achromatic control (white) and resting‐state (black, no built environment) significantly increased the range in respiration and skin conductance response. We also detected a significant increase in alpha frontal midline power and frontal hemispheric lateralization relative to blue condition, and increased power spectral density across all electrodes in the blue condition for theta, alpha, and beta bandwidths. The ability for built environment design to modulate emotional response has the potential to deliver significant public health, economic, and social benefits to the entire community. The findings show that blue coloring of the built environment increases autonomic range and is associated with modulations of brain activity linked to emotional processing.

**Impact Statement**

Our empirical study provides evidence that blue color in the built environment modulates oscillatory and autonomic activity linked to emotional processing. We measured neural correlates and physiological indicators during exposure to color of an enclosed interior room. This involved using an immersive Cave Automatic Virtual Environment and monitoring indoor environmental quality variables responsible for physiological comfort. This research and novel technique will help us understand how to design interior spaces for optimal mental health.

## 1 INTRODUCTION

We have a limited understanding of the impact that built environment exposure has on our mental health (Anderson et al., 2018; Hoisington et al., 2019). Early life studies have shown the importance of environmental experience in shaping brain development (McLaughlin et al., 2014). In observational studies of individuals deprived of sensory input in their physical and social environment (imprisonment, orphanages, etc.), participants demonstrate permanent alterations in brain structure and function (Mackes et al., 2020). Similarly, environmental enrichment studies in animal models demonstrate the substantial role that environment has on cellular, molecular and behavioral systems (Nithianantharajah & Hannan, 2006). An increasing number of studies are investigating the public health implications of built environment design. This includes functional properties (enabling movement and facilitating activities) (Benton et al., 2016; Kärmeniemi et al., 2018; Pinter‐Wollman et al., 2018) and indoor environmental qualities (Tham et al., 2020). However, we are only beginning to disentangle and rigorously assess the impact of design characteristics such as color, texture, geometry, and scale (Bower et al., 2019). The novelty of social and physical environmental exposures inherent in daily life, alongside the ethical limitations of controlling environmental exposures over prolonged periods of human development, makes this difficult to investigate, with the relatively few studies conducted to date providing only weak evidence (Moore et al., 2018). Nevertheless, through isolating design characteristics of built environments and understanding confounding variables, we can begin to interrogate the impact our surroundings have on our neurophysiological response and, subsequently, mental health.

Color is an integral component of perception (Gegenfurtner, 2003) and has been recognized as an important design characteristic within built environments (Caivano, 2006). Spatially, it can distinguish areas for purpose, aid navigation (Jansen‐Osmann & Wiedenbauer, 2004), guide behavior through social and contextual associations (Maier et al., 2009), and create illusionary distortions to our perception (Corney et al., 2009; Guibal & Dresp, 2004; von Castell, Hecht, & Oberfeld, 2018b). While in popular culture it has been suggested that color hue has direct effects on emotion, these associations have been linked to variations in lightness and chroma rather than hue (Schloss et al., 2020). A difficulty in synthesizing results from different studies is caused by the variety of color sources used. Studies conducted in relation to the impact of color on brain activity, as measured using electroencephalography (EEG) and/or physiological response in humans include those which use a light source: halogen, incandescent (Park et al., 2013), light‐emitting diode (LED) (Chinazzo et al., 2020; Stamps, 2010); or ink‐based source: ambient color (painted surfaces) (Ainsworth et al., 1993; von Castell, Stelzmann, et al., 2018), and stimulus color (dyed or printed material) (Gao & Xin, 2006; Yoto et al., 2007). Furthermore, studies have either explored the individual dimensions of color: Chroma (saturation) (Dresp‐Langley & Reeves, 2014; Zieliński, 2016), hue (pigmentation) (Mehta & Zhu, 2009), and value (brightness or darkness adjustment) (Knez, 2001; von Castell, Hecht, & Oberfeld, 2018a), or used an approach combining and reporting variations in dimensions. When the color is generated through a light source, color temperature is also measured to describe the characteristic of the visible light (Kraneburg et al., 2017; Lasauskaite & Cajochen, 2018). However, there remains difficulty understanding color perception due to variables such as surface materiality, geometry, light field structure, and the viewing position of the observer (Marlow et al., 2012), with research further indicating differences between ink‐based and light‐source color (Lee et al., 2020). Adding to the difficulty, when investigating the impact of color in the built environment, several recent virtual reality studies have used context‐rich settings (bedroom, classroom, hospital waiting room) and have not controlled for indoor environmental qualities (IEQ) outside of the virtually presented environment (Cha et al., 2020; Lipson‐Smith et al., 2020; Llinares et al., 2021). With the complexity of dimensions and influencing variables, it has been recognized that applied domains, such as architecture, frequently make assumptions on the effect of color on emotion, without adequately controlling and reporting conditions in the studies (Elliot, 2015; Wilms & Oberfeld, 2018).

Our association with color is also influenced by the intrinsic properties of light and the human visual system. Visual perception of color results from different power spectral distributions of light wavelengths and illumination levels of the environment, which modulate retinal photoreceptor response. As a result of these interacting variables, blue is harder for the human eye to focus on during high illumination levels (i.e., daylight and indoor lighting settings). Consequently, blue is less frequently used for signage and signaling where behavioral action and/or inhibition are critical for safety during daylight. The interaction of both biological and contextual learned responses are known as color‐in‐context theory (Elliot & Maier, 2012). Studies that explore our conditioning to color in signage have demonstrated blue is less likely to affect behavioral compliance (Braun & Silver, 1995). Similarly, when applied to the built environment, studies examining color‐emotion associations within interior built environments have found that ‘neutral’ is the most‐stated self‐report to the color blue, compared to red, green, and gray, which invoke a range of emotional associations (Güneş & Olguntürk, 2020). As we cannot remove the social conditioning we experience to colors in our environment, we selected blue as a ‘neutral’ associated chroma to interrogate.

There is limited research in this field, and results remain highly variable, with no accepted standards of practice and/or conclusive meta‐analysis currently available to understand the impact of built environment color on emotional and neurophysiological response. However, techniques to measure autonomic activity such as heart rate variability (HRV), respiration, and skin conductance response (SCR), can enable us to objectively understand the emotional experience. Physiological findings from the impact of blue color on emotion are inconsistent, with mixed findings on whether HRV is increased with blue color (Küller et al., 2009), decreased (AL‐Ayash et al., 2016), or unaffected (Jacobs & Hustmyer, 1974). Similarly, it is unclear what effect blue has on SCR, with contradictory findings on whether an effect exists; and where an effect is found, disparity in whether it results in a positive or negative response (Mikellides, 1990; Rajae‐Joordens, 2011). Reduced HRV has been suggested as an indicator of less favorable health, with an increased risk of chronic heart disease (Dekker et al., 2000). Similarly, greater variability in respiration and skin conductance response range are associated with higher levels of arousal and anxiety (Chattopadhyay et al., 1975; Masaoka & Homma, 1997), and increased physiological arousal is associated with anxiety, which can have detrimental effects on cognitive performance (Maloney et al., 2014). Consequently, if we could optimize autonomic responses through color in the built environment, we may be able to support these conditions. Another non‐invasive technique that can be used to understand the impact of color is EEG. EEG is a non‐invasive technique that measures neural oscillations, enabling us to investigate modulations in brain activity. Emerging research indicates that EEG can be used to classify and detect color stimuli (Göksel Duru & Alobaidi, 2021), and that color can influence attentional capture (Wu et al., 2020), however, the link between color and emotion is yet to be established. Studies investigating emotion using EEG have found frontal midline power (Aftanas & Golocheikine, 2001) and frontal hemispheric lateralization can be used as neurophysiological correlates. Specifically, lower alpha and theta power in the left‐ than right‐hemisphere is associated with positive emotion, while lower power in the right‐ than left‐hemisphere can be seen for negative emotion (Coan & Allen, 2004; Davidson, 2004).

**FIGURE 1:**
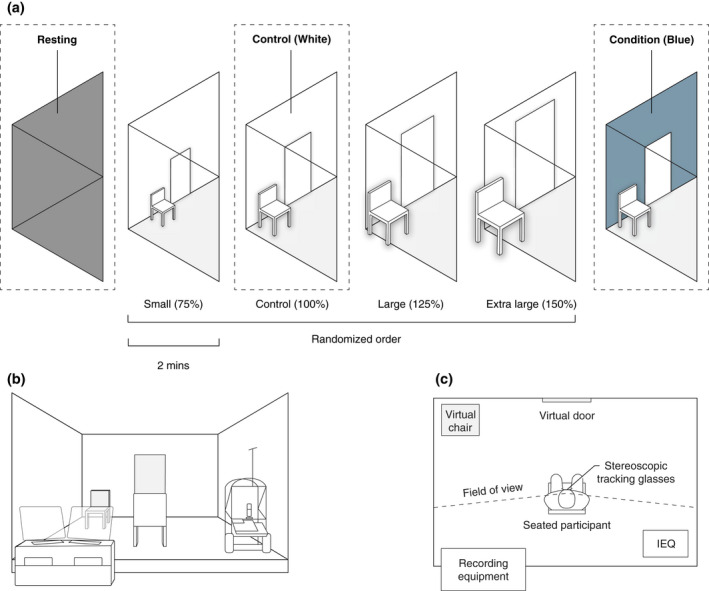
Experimental design and setup. (a) Isometric cut away of conditions (including the three scale conditions not included in this study), (b) one‐point perspective of the mixed reality environment showing virtual features in gray (chair, door) and the physical environment and equipment in white. (c) Floor plan indicating the position of items in the experiment. Participants were presented with eyes open resting state, followed by four randomized scale conditions (not analyzed in this paper). In this study, we analyzed the blue condition which was presented after the scale conditions in conjunction with the black resting state and white control condition. Each scene lasted two‐minutes, between which the resting state was displayed while the participant completed a self‐report assessment of emotion. Indoor environmental quality (IEQ) variables were measured continuously, and all recording equipment was positioned outside of the participants' field of view. Diagrams are representative, not drawn to exact scale.

In this study, we investigated whether the blue ambient wall color of an interior room would result in modulation of autonomic, EEG, and self‐report indicators of emotion. We selected blue due to previous studies indicating it invokes the most neutral self‐report of emotion association. A Cave Automatic Virtual Environment (CAVE) was used to create an environmentally controlled and cost‐effective simulation, enabling greater sensorimotor integration than virtual reality headsets (Kalantari et al., 2021; Sanchez‐Vives & Slater, 2005). Chromatic adaptation was avoided through the achromatic black resting state prior to exposure (Jameson et al., 1979). We selected the color blue and matched the properties to a popular paint sample from a large local paint manufacturer to achieve greater ecological validity of a color sample we are exposed to in built settings. The virtual built environment included visual cues in the form of a closed door and a chair within the scene to help participants determine the height, width, and surface depth (Brouwer et al., 2005) (Figure 1). We also collected participant socio‐demographic and personality data to understand if characteristics in the study sample interacted with the results. We expected the visually salient nature of the color condition would result in increased autonomic response (Zieliński, 2016), and higher frontal and occipital activation in the theta and alpha bandwidths, as these are associated with emotion (Aftanas & Golocheikine, 2001; Coan & Allen, 2004; Davidson, 1992). Due to the neutral color‐emotion association, we expected self‐report of pleasure and dominance would remain consistent while arousal would be increased.

## 2 RESEARCH DESIGN AND METHOD

The study was approved by the Deakin University Human Research Ethics Committee and carried out in accordance with relevant guidelines and regulations. All participants provided informed and written consent. Participants were offered a $20.00 AUD gift voucher as reimbursement for their time.

### 2.1 Participants

Eighteen right‐handed adult participants aged between 18 and 55 years old (8 women, mean age = 34.5 ± 9.87 years) were recruited for this study via university and community advertisements. This was a subset of the participants from a study investigating scale. Due to the experimental nature of the study and to reduce confounding variables we selected a healthy adult sample. To be included, participants had to meet the following criteria: (1) Normal or corrected‐to‐normal vision, (2) able to speak and read English, (3) no prior psychiatric, neurological, or neurodevelopmental conditions, and (4) no prior experience or training in built environment design.

### 2.2 Procedure

The study took place over a one‐week period at Deakin University Waurn Ponds Campus, Geelong, Victoria, Australia. Each session ran for approximately 90‐min, and participants were tested individually. On arrival, participants completed demographics and personality questionnaires. Participants were next fitted with the equipment prior to being brought into the experiment room. Skin surfaces were cleaned before fitting physiological equipment and a 64‐channel cap (Philips Hydrocel Geodesic Sensor Net 64‐channel HCGSN) was used for acquiring EEG data. The cap was soaked in an electrolyte solution and towels were placed over the participants' lap and shoulders for comfort and to protect the cords before the cap was fitted. EEG data were recorded using Net Station 5 Geodesic EEG software, version 5.4.2 (Electrical Geodesics Inc), and acquired at a sampling rate of 1000 Hz, with Cz as the online reference. A continuous recording was created for each participant and was manually time‐stamped by the researcher at the start and end of each 2‐min scene exposure using markers. Most impedances were kept under 50 kΩ (considered an acceptable level of impedance for this system) with an average value of 25.2 kΩ (*SD* = 7.75).

Participants were seated in the CAVE for a total duration of approximately 15–20 min to complete the experiment. No natural light was present in the experimental room, overhead lights were turned off during the experiment and participants were tested within daylight hours. Previous studies have suggested that time and duration of blue light exposure can modulate cardiovascular physiological effects (Chellappa et al., 2017). This has thought to be linked to the endogenous circadian timing system, which can trigger changes in blood pressure, vascular tone, heart rate (HR), and variation in beat‐to‐beat intervals such as SDRR and RMSSD (Boudreau et al., 2012). We conducted an a‐posteriori analysis on time of day to confirm this did not influence responsivity to color (see Supporting Information).

We elected to run a static resting‐state study where participants sat immersed in the space, rather than setting a task and/or involving physical movement, as this has been thought to affect cognition and memory (Pettijohn & Radvansky, 2016). The seated position mitigated for participant height variability and helped minimize artifact in the electrophysiological measures. The rationale for the static resting‐state technique is that isolating design elements will enable further understanding of which features within the built environment make a difference before they can be combined to understand the effect of multiple design characteristics. Each session began with a resting state recording with eyes open to establish baseline. Participants were then exposed to six scenes displayed for two‐minutes each in randomized order, using a generated plan for 100 subjects in one block (seed 28107). Three of these scenes are not included in this paper as they were related to modulating room‐scale. A period of two‐minutes per condition was selected to ensure sufficient EEG data to epoch for stability in connectivity analysis (van Diessen et al., 2015), while avoiding habituation from the experimental conditions and nature of the scenes that could confound the results (O'Gorman, 1977). The two‐minute recording period is not uncommon for this type of study (Rogala et al., 2020; Vellante et al., 2020). At the end of each scene, the virtual environment was returned to the resting state scene and the participant was asked to complete a short self‐report survey using a 5‐point visual Self‐Assessment Manikin (Bradley & Lang, 1994; Mehrabian, 1996). During the scene the researcher was outside of the CAVE, monitoring data quality in the recordings.

### 2.3 Equipment and stimuli

#### 2.3.1 Cave automatic virtual environment

The CAVE consisted of three walls (3 m wide × 2.4 m high) and a floor (2.4 m wide × 3 m long), each with Barco Galaxy NW‐12 stereoscopic projectors connected to a series of image generators (computers) with Nvidia Quadro P6000 graphics cards. Quadro Sync II cards at 120 Hz (60 Hz per eye) were used to frame lock the projectors to ensure rendered images were displayed at the same time. An optical‐based tracking system consisting of eight cameras tracked user movements within the CAVE to LED markers located on the stereoscopic glasses. The tracking system operates at 240 Hz with sub‐millimeter accuracy and connects back to a Virtual Reality Peripheral Network (VRPN) server. The CAVE uses a custom‐built Unity environment to run VR experiences with Vertical Sync (VSync) set to 60 frames per second. The unity environment connects to the tracking systems via VRPN server using an ethernet connection and updates the tracked position on each rendered frame.

#### 2.3.2 Virtual environment development and CAVE integration

We created a 3D model that represented a conventional cubic room in Autodesk Revit that was then exported into the Unity game engine (2019.2.15) for CAVE integration. We next applied textures, using a matte plaster texture to the three wall surfaces, a slight gloss texture of bumpy concrete to the floor, a matte wood texture to the door, doorway, and chair, and a low gloss metal surface applied to the door handle. Once material color, texture settings, and lighting had been applied to the model, the room was duplicated (Unity Prefabs) into separate rooms to represent the conditions. Pre‐baked lightmaps were applied for each scaled room to ensure consistent lighting and texture relative to the scale and ‘realistic’ as possible to view.

The built environment conditions were designed using Standards Australia measurements for a residential internal door for reference scale (820 mm × 35 mm × 2040 mm) (Standards Australia, 2017), and room dimensions were modeled of the physical CAVE walls (3200 mm × 3200 mm × 2400 mm). The resting‐state scene (no built environment) was rendered in black (R0, G0, B0, hue (degrees) = 0, saturation (%) = 0, brightness (%) = 0. However, due to white finish of the projector screens, while we created a virtual scene with a black background, this appears as a dark gray when displayed on the screens. In the control (white) condition, the wall surfaces were rendered with a white finish (R255, G255, B255, hue (degrees) = 0, saturation (%) = 0, and brightness (%) = 100, and smoothness = 50%). For a realistic color scene, the condition (blue) wall surfaces were rendered with Dulux ‘Post Boy A359’ (R122, G155, B173, hue (degrees) = 198, saturation (%) = 35.3, brightness (%) = 67.8, and smoothness = 50%). Data on the indoor environmental quality (IEQ) were collected throughout the study to ensure any fluctuations to these properties linked to data which may influence emotion and neurophysiological response were minimized.

#### 2.3.3 Indoor environmental quality

We acquired data using CR100 Measurement and Control System with LoggerNet 4.6.2 software (Campbell Scientific, Inc). A test recording was completed prior to the experiment to calibrate the equipment, ensuring the readings were accurate in accordance with EN ISO 7730 Fanger Comfort Model (Fanger, 1970).

We recorded IEQ data at 1‐min intervals which were date and time‐stamped. We averaged the 1‐min readings from the corresponding time stamped data within each participants' session to create an overall average per person and then determined the average across all participants. Although the VR lab was acoustically soundproof and no talking occurred during the scene recordings, a handheld sound level meter was used to capture fluctuating mechanical equipment noises from the CAVE projector lamp ventilation and cooling system which could not be controlled. Sound level recordings were conducted at different intervals during experiments to establish an overall range across the 5‐week data‐collection period. Overall mean air and wet‐bulb globe temperature was within the 21–25°C range for optimal performance (Seppänen & Fisk, 2006), the carbon dioxide concentration throughout the testing period was within the indoor air concentration range of 500–1500 ppm, and the mean relative humidity was under 50% (Seppänen & Fisk, 2004). Sound pressure levels were also within an accepted range for the experiment (Basner et al., 2014).

### 2.4 Data analysis

#### 2.4.1 Physiological data

We acquired physiological data using PowerLab 4/35 (ADI Instruments PL3504) with a respiratory belt transducer (ADI Instruments TN1132/ST), Ag/AgCl ECG electrodes (Ambu Bluesensor N), and SCR finger plate electrodes (ADI Instruments MLT118F). Data for all physiological measures were acquired at 1000 Hz, circuit zero was applied before the first recording and a subject zero was undertaken between each condition recording. Online filtering parameters differed between measures: ECG −100 to 100 mV; SCR −40 to 40 μS; and respiration −10 to 10 V. Five channels were set to record and calculate ECG, SCR and respiration.

For HRV, we analyzed the RMSSD and SDRR time domain components of the QRS complex within the ECG recording in accordance with the Task Force of the European Society of Cardiology and the North American Society of Pacing and Electrophysiology (Camm et al., 1996). Ectopic heartbeats were excluded from the analysis. We adjusted detection to a minimum peak height of 1.2 *SD* and typical QRS width between 80 ms over a 350 ms minimum period. Beat classification for RR intervals was set between 600 and 1400 ms with a complexity of 1 to 1.5. We also applied a low‐pass filter of 30 Hz. We used the cyclic measurements function for respiration frequency with scoring parameters of 1.3 standard deviation threshold for detecting minimum peak height. Due to time lag in‐breath detection at the beginning of the recording, the first and final 7 s of the continuous file for each participant across conditions were removed.

Finally, we extracted physiological data using LabChart Pro 8.1.16 (AD Instruments) and analyzed the results using RStudio (Version 1.3.959). To correct for distribution, a log transformation (log10) was applied to the physiological data. A within subjects repeated measures ANOVA with the Greenhouse–Geisser (G‐G) correction for sphericity was used across the measures we analyzed. To control for multiple comparisons, the False Discovery Rate (FDR) method was applied to the results (Benjamini & Hochberg, 1995).

#### 2.4.2 EEG data

Pre‐processing was conducted using the EEGLab plugin (v2019.1) (Delorme & Makeig, 2004) for MATLAB R2019b (v9.7.0.1471314, MathWorks, Inc). Continuous EEG data were bandpass filtered from 1 to 70 Hz (zero‐phase Butterworth filter) in EEGLab. To exclude electrical interference from the CAVE environment we applied a 47 to 53 Hz notch‐filter. We removed eye channels and the Cz reference channel. We then rejected channels if the kurtosis value was >5 standard deviations outside the average and replaced information in those channels using a spherical spline interpolation. We then re‐referenced data to the common average reference of all channels. Next, we applied the SOUND algorithm to aid in the removal of recording noise using input parameters of five iterations to evaluate noise in each channel and 0.2 regularization level (lambda value) to control the amount of cleaning (Mutanen et al., 2018). Independent component analysis (FastICA algorithm) was then performed (Hyvärinen & Oja, 2000), with artifactual components identified with assistance from the ICLabel plugin (Pion‐Tonachini et al., 2019). A component was removed if the probability of that component containing brain data was less than 30% and the component was not in the ‘other’ category.

To separate the data into conditions, we used event markers to split each continuous file from the participant into 120 second block files using the start marker for each condition. We then segmented the data into three‐second epochs, resulting in 40 epochs per participant. We then performed additional artifact rejection to remove any data exceeding ±150 μV using the EEGLab ‘*pop_eegthresh*’ function. To check on data quality, we calculated the average epochs remaining after cleaning for each condition and participant (mean epoch = 39.7, ±.535). Finally, data from each participant/electrode were converted to the frequency domain using the Fast Fourier Transform (FFT) with Hanning taper in the FieldTrip toolbox for EEG/MEG‐analysis (1 Hz frequency steps between 1 and 70 Hz) (Oostenveld et al., 2011). We then created averages across each separate frequency band for each electrode: delta (1–3 Hz), theta (4–7 Hz) alpha (8–12 Hz), beta (13–29 Hz), low gamma (30–45 Hz), and high gamma (55–70 Hz). Power was then averaged over electrodes within three a priori regions of interest: frontal midline (AFz, Fz, FCz), frontal right‐hemispheric sites (F10, F8, AF4, F6, FT8, F2, F4, FC6, FC4, and FC2), and frontal left‐hemispheric sites (F9, F7, AF3, F5, FT7, F1, F3, FC5, FC, and FC1). To understand the hemispheric difference, we generated a lateralization index (Thut et al., 2006) to understand the difference between the average over the frontal left and right regions of interest:

$$\text{(α)}={\text{(α}}^{“}{\left(\text{right}\right)}^{”}-{\text{α “(left)”)/(α}}^{“}{\left(\text{left}\right)}^{”}+{\text{α}}^{“}{\left(\text{right}\right)}^{”})$$

Using this index, higher values correspond to stronger power in the right than the left hemisphere.

Lastly, we applied a log transform (log10) to correct for distribution. We removed outliers that caused the violation of normality assumptions (according to the Shapiro–Wilk test) prior to statistical analysis. A within subjects repeated measures ANOVA with G‐G correction was used across the EEG data. To correct for multiple comparisons where significance was detected within subjects, the false discovery rate (FDR) method was applied to post‐hoc comparisons (Benjamini & Hochberg, 1995).

## 3 RESULTS

### 3.1 Overview

To study the effects of room color on physiological responses, we examined the mean and range indices of heart rate/variability, skin conductance response, and respiration. We investigated the power spectra, frontal lateralization, and midline power in the alpha and theta bandwidths. For completeness, the power‐spectra of the remaining bandwidths are presented in Tables 1 and 2. We also investigated self‐report responses compared to physiological and EEG responses to determine if there was a relationship.

**TABLE 1:** Descriptives (mean/standard deviation) for physiological analysis

Condition	Resting	Control (white)	Condition (blue)
HRV RMSSD (log10) mean/*SD*	1.58/.235	1.53/.209	1.52/.207
HRV SDRR (log10) mean/*SD*	1.69/.158	1.66/.151	1.66/.145
Resp mean (log10) mean/*SD*	1.26/.093	1.25/.087	1.24/.142
Resp mx‐mn (log10) mean/*SD*	.130/.078	.042/.146	1.30/.361
SCR mean/*SD*	−.104/7.00	−.303/2.18	.225/3.09
SCR mx‐mn (log10 + 1) mean/*SD*	.220/.085	.198/.089	.638/.256

**TABLE 2:** Statistical significance values for physiological measures

Condition	Resting		Control (white)
Comparison	Control (white)	Condition (blue)	Condition (blue)
HRV RMSSD (log10) *P*/*P* ^FDR^	.026*/.044*	.029*/.044*	.489/.489
HRV SDRR (log10) *P*/*P* ^FDR^	.236/.708	.485/.728	.777/.777
Resp mean (log10) *P*/*P* ^FDR^	.516/.955	.955/.955	.757/.955
Resp mx‐mn (log10) *P*/*P* ^FDR^	.008**/.008**	<.001***/<.001***	<.001***/<.001***
SCR mean *P*/*P* ^FDR^	.903/.903	.851/.903	.537/.903
SCR mx‐mn (log10 + 1) *P*/*P* ^FDR^	.599/.599	<.001***/<.001	<.001/<.001***

*Note*: Statistics are derived from one‐way repeated measures ANOVA's. *P* = *p* value, ^FDR^ = false discovery rate correction.

*p ≤ .05, **p ≤ .01, ***p ≤ .001.

### 3.2 Blue colored room increases autonomic variability in skin conductance response and respiration

SCR range (i.e., maximum minus minimum slope) significantly differed across the resting state, white control and blue condition [*F*(1, 18) = 71, *p* = <.001, ${\eta^{2}}_{p}$ = .845]. Post‐hoc comparisons showed that the blue (condition) room range was greater than the resting state [*M*_diff_ = −.491, *SE*_diff_ = .056, *t*(13.0) = −8.839, *p*_corrected_ = <.001, 95% CI (−.612, −.371)], and also significantly greater than the white (control) room [*M*_diff_ = −.505, *SE*_diff_ = .056, *t*(13.0) = −8.994, *p*_corrected_ = <.001, 95% CI (−.581, −.360)]. There was no difference between the resting state and white control [*M*_diff_ = .015, *SE*_diff_ = .028, *t*(13.0) = −.085, *p*_corrected_ = .599, 95% CI (−.045, .074)]. We did not detect an effect of condition on mean SCR [*F*(1, 23) = .068, *p* = .869, ${\eta^{2}}_{p}$ = .004].

A similar pattern was observed in respiration where we did not detect an effect of condition for the mean respiration rate [*F*(1, 18) = .071, *p* = .833, ${\eta^{2}}_{p}$ = .005], but there was an effect of condition for the Mx‐Mn slope [*F*(1, 20) = 199, *p* = <.001, ${\eta^{2}}_{p}$ = .921]. Follow‐up analyses indicated that respiration range increased for the blue condition when compared to the white control [*M*_diff_ = −1.258, *SE*_diff_ = .085, *t*(17.0) = −14.77, *p*_corrected_ = <.001, 95% CI (−1.438, −1.078)], and when compared to the resting state [*M*_diff_ = −1.170, *SE*_diff_ = .082, *t*(17.0) = −14.28, *p*_corrected_ = <.001, 95% CI (−1.343, −.997)]. Respiration range decreased for the white control when compared to the resting state [*M*_diff_ = .088, *SE*_diff_ = .030, *t*(17.0) = 2.98, *p*_corrected_ = .008, 95% CI (.026, 1.50)].

An effect of condition was seen in the root mean square successive difference (RMSSD), our primary measure of heart rate variability [*F*(2, 27) = 4.45, *p* = .026, ${\eta^{2}}_{p}$ = .218]. Post‐hoc analysis showed RMSSD decreased in the blue condition compared to the resting state [*M*_diff_ = .058, *SE*_diff_ = .024, *t*(16.0) = 2.390, *p*_corrected_ = .044, 95% CI (.007, .109)], and also decreased for the white control compared to the resting state [*M*_diff_ = .045, *SE*_diff_ = .018, *t*(16.0) = 2.456, *p*_corrected_ = .044, 95% CI (.006, .084)]. We found no difference between the white control and blue condition [*M*_diff_ = .013, *SE*_diff_ = .018, *t*(16.0) = .707, *p*_corrected_ = .489, 95% CI (−.025, .051)].

**FIGURE 2:**
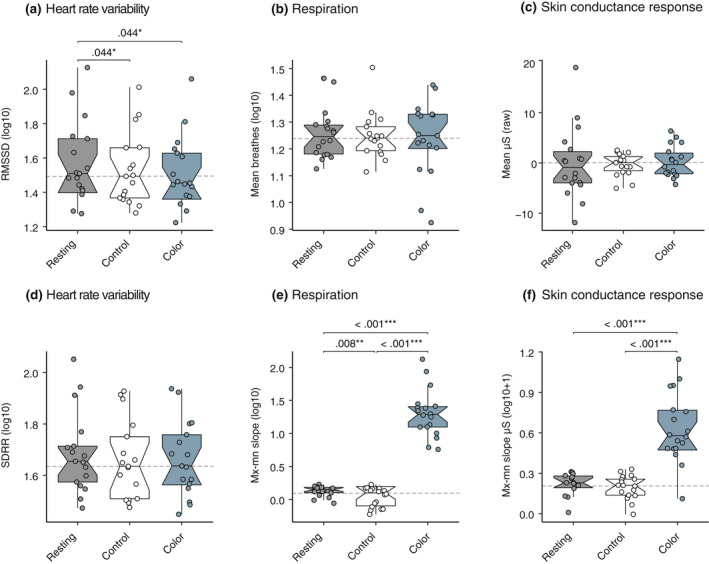
Physiological measures between the resting‐state, control, and color condition. (a–f) boxplots with quartile ranges and medians for physiological measures analyzed using transformed values with outliers removed. Each data point represents a participant's averaged response from the 2‐min exposure. Significance values (FDR‐corrected) from the data after transform and removal of outliers have been superimposed to indicate where significant differences were found. All participants were exposed to the resting state first, before the control (white) and condition (blue). Mean values did not show an effect, however, measures analyzing the change in range, such as maximum—minimum slope for skin conductance response and respiration, showed differences.

No effect of condition was found for heart rate variability measured by the standard deviation of the R‐R interval (SDRR) [*F*(2, 26) = .678, *p* = .448, ${\eta^{2}}_{p}$ = .041]. Results are shown in Figure 2, with descriptives and *p*‐values for each comparison presented in Tables 1 and 2.

### 3.3 Correlates of emotional response were detected in the alpha bandwidth for frontal midline power and frontal hemispheric lateralization

To investigate emotion‐related EEG results, we compared the frontal midline power and frontal lateralization in theta and alpha bandwidths. An effect of condition was seen in the frontal midline power in the alpha bandwidth [*F*(1, 22) = 4.09, *p* = .047, ${\eta^{2}}_{p}$ = .194]. Post hoc analysis showed that alpha midline power was increased in the blue condition in comparison to the control [*M*_diff_ = −.093, *SE*_diff_ = .0024, *t*(17.0) = −3.874, *p*_corrected_ = .003, 95% CI (−.143, −.042)]. There was no significant difference between the resting state and blue condition [*M*_diff_ = .041, *SE*_diff_ = .054, *t*(17.0) = .760, *p*_corrected_ = .458, 95% CI (−.074, .156)], or resting state to the white control [*M*_diff_ = .134, *SE*_diff_ = .058, *t*(17.0) = 2.307, *p*_corrected_ = .051, 95% CI (.011, .257)]. We did not detect an effect for theta midline power [F(2, 30) = 2.27, *p* = .125, ${\eta^{2}}_{p}$ = .118].

**FIGURE 3:**
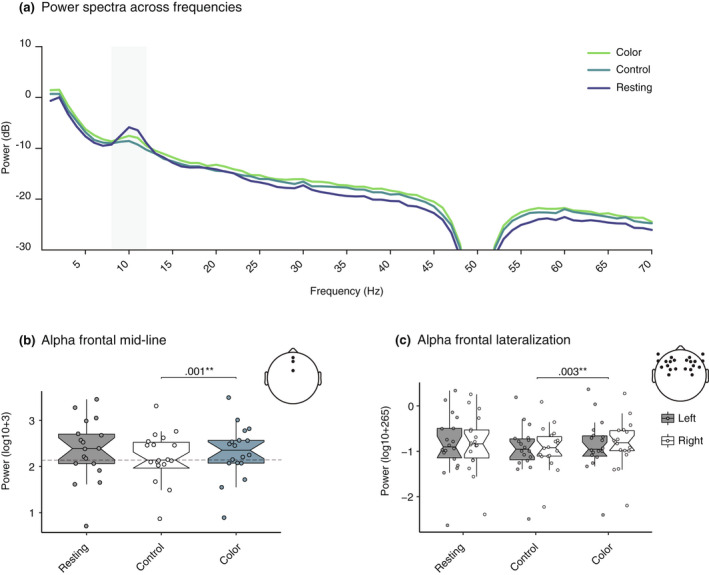
Significant differences in EEG alpha frontal midline power and frontal hemispheric lateralization were found. (a) Power spectra plot showing power (dB) across frequencies. The alpha bandwidth (8 to 12 Hz) is highlighted with the gray shading box. The dip represents the 47–53 Hz notch filter applied to remove electrical interference from the CAVE environment. (b) Boxplot with quartile ranges and medians to show increased alpha midline power spectra and frontal lateralization. (c) Hemisphere (gray for left, white for right) with significance overlaid from calculating the lateralization index showing increased left hemispheric lateralization in the alpha bandwidth.

An effect was detected in frontal lateralization within the alpha bandwidth [*F*(2, 24) = 9.34, *p* = .002, ${\eta^{2}}_{p}$ = .384]. Post‐hoc analysis revealed there was significantly greater lateralization, with higher left side amplitude over the right, in the blue condition relative to the white control [*M*_diff_ = −.034, *SE*_diff_ = .015, *t*(16.0) = −2.25, *p*_corrected_ = .003, 95% CI (−.067, −.006)]. We also found greater lateralization in the white control, compared to the resting state [*M*_diff_ = .051, *SE*_diff_ = .021, *t*(16.0) = −2.45, *p*_corrected_ = .059, 95% CI (.007, .096)], however this did not survive correction. We did not detect an effect for lateralization in the theta bandwidth [*F*(2, 27) = 3.19, *p* = .058, ${\eta^{2}}_{p}$ = .186]. Figure 3 illustrates the power spectra across frequencies and the alpha frontal midline and lateralization results. Descriptives and *p*‐values for each comparison are presented in Tables 1 and 2.

### 3.4 Significant differences in power spectral density in the theta, alpha, and beta bandwidths between white and blue room conditions were detected

We found a significant effect across the averaged power spectra for theta, alpha and beta bandwidths. An effect was detected in theta amplitude [*F*(2, 31) = 12.6, *p* = < .001, ${\eta^{2}}_{p}$ = .425]. Follow‐up analysis indicated that theta amplitude was increased for the white control when compared to the resting state [*M*_diff_ = −.068, *SE*_diff_ = .027, *t*(17.0) = −2.49, *p*_corrected_ = .024, 95% CI (−.126, −.010)], increased for the blue condition when compared to the resting state [*M*_diff_ = −.126, *SE*_diff_ = .026, *t*(17.0) = −4.78, *p*_corrected_ = <.001, 95% CI (−.181, −.070)], and increased for the blue condition when compared to the white control [*M*_diff_ = −.058, *SE*_diff_ = .021, *t*(17.0) = −2.73, *p*_corrected_ = < .001, 95% CI (−.102, −.013)]. Alpha changes were also detected [*F*(1, 23) = 3.52, *p* = .062, ${\eta^{2}}_{p}$ = .172]. Alpha amplitude decreased for the blue condition when compared to the white control [*M*_diff_ = −.091, *SE*_diff_ = .028, *t*(17.0) = −3.200, *p*_corrected_ = .015, 95% CI (−.150, −.031)]. There was also an effect on beta activity [*F*(2, 25) = 10.5, *p* = <.001, ${\eta^{2}}_{p}$ = .412]. Post‐hoc comparisons showed that there was a significant decrease in amplitude during the blue condition when compared to the resting state [*M*_diff_ = −.132, *SE*_diff_ = .032, *t*(15.0) = −4.16, *p*_corrected_ = <.001, 95% CI (−.200, −.064)], a decrease during the white control when compared to the resting state [*M*_diff_ = −.074, *SE*_diff_ = .032, *t*(15.0) = −2.29, *p*_corrected_ = .037, 95% CI (−.142, −.005)], and a decrease during the blue condition in comparison to the white control [*M*_diff_ = −.058, *SE*_diff_ = .022, *t*(15.0) = −2.72, *p*_corrected_ = .024, 95% CI (−.104, −.013)]. Although we detected differences within the remaining bandwidths, follow‐up analysis revealed these were not between the white control and blue condition. This included delta [*F*(1, 30) = .004, *p* = .370, ${\eta^{2}}_{p}$ = .292], low gamma [*F*(2, 28) = 6.46, *p* = .007, ${\eta^{2}}_{p}$ = .275], and high gamma [*F*(2, 30) = 4.61, *p* = .022, ${\eta^{2}}_{p}$ = .213] amplitudes.

**FIGURE 4:**
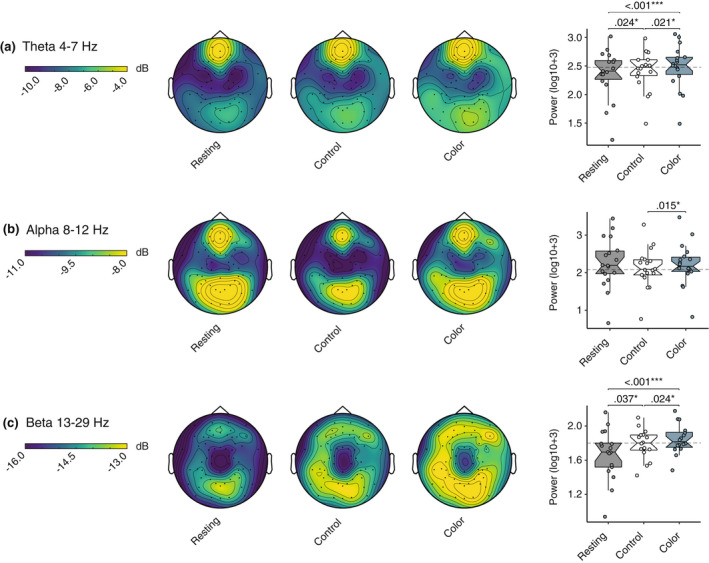
Significant differences in EEG power spectral density in theta, alpha, and beta bandwidths were detected. To illustrate the differences, we have plotted EEG topographies and boxplots with quartile ranges and medians for the overall power spectral density in the theta, alpha, and beta bandwidths. (a) Theta 4 to 7 Hz with amplitude range of −10 to −4 decibels. (b) Alpha 8 to 12 Hz with an amplitude range −11 to −8 decibels. (c) Beta 13 to 29 Hz with an amplitude range −16 to −13 decibels.

Results are shown in Figure 4, with descriptives and *p*‐values for each comparison presented in Tables 3 and 4.

**TABLE 3:** Descriptives (mean/standard deviation) for EEG analysis

Condition	Resting	Control (white)	Condition (blue)
Power spectral density (log10)			
Delta 1 to 3 Hz (log10 + 3) mean/*SD*	3.03/.456	3.11/.384	3.16/.455
Theta 4 to 7 Hz (log10 + 3) mean/*SD*	2.36/.431	2.42/.346	2.48/.383
Alpha 8 to 12 Hz (log10 + 3) mean/*SD*	2.26/.661	2.13/.528	2.22/.565
Beta 13 to 29 Hz (log10 + 3) mean/*SD*	1.67/.297	1.79/.177	1.84/.172
Low Gamma 30 to 45 (log10 + 3) mean/*SD*	1.22/.300	1.35/.369	1.39/.342
High Gamma 55 to 70 (log10 + 3) mean/*SD*	.769/.356	.922/.418	.962/.384
Frontal hemispheric lateralization			
Theta 4 to 7 Hz (log10 + 200) mean/*SD*	2.20/.164	2.27/.092	2.24/.183
Alpha 8 to 12 Hz (log10 + 265) mean/*SD*	2.33/.184	2.25/.192	2.29/.195
Frontal midline power spectral density			
Theta 4 to 7 Hz (log10 + 3) mean/*SD*	2.75/.437	2.78/.481	2.36/.660
Alpha 8 to 12 Hz (log10 + 3) mean/*SD*	2.36/.660	2.22/.538	2.32/.581

**TABLE 4:** Statistical significance values for EEG analysis

Condition	Resting		Control (white)
Comparison	Control (white)	Condition (blue)	Condition (blue)
Power spectral density (log10)			
Delta 1 to 3 Hz (log10 + 3) *P*/*P* ^FDR^	.060/.073	.003**/.009**	.073/.073
Theta 4 to 7 Hz (log10 + 3) *P*/*P* ^FDR^	.024*/.024*	<.001***/<.001***	.014*/.021*
Alpha 8 to 12 Hz (log10 + 3) *P*/*P* ^FDR^	.040*/.060	.474/.474	.005**/.015*
Beta 13 to 29 Hz (log10 + 3) *P*/*P* ^FDR^	.037*/.037*	<.001***/.001**	.016*/.024*
Low Gamma 30 to 45 (log10 + 3) *P*/*P* ^FDR^	.021*/.032*	.009**/.027*	.272/.272
High Gamma 55 to 70 (log10 + 3) *P*/*P* ^FDR^	.019*/.038*	.025*/.038*	.524/.524
Frontal hemispheric lateralization			
Theta 4 to 7 Hz (log10 + 200) *P*/*P* ^FDR^	.070/.105	.042*/.105	.576/.576
Alpha 8 to 12 Hz (log10 + 265) *P*/*P* ^FDR^	.034*/.051	.458/.458	.001**/.003**
Frontal midline power spectral density			
Theta 4 to 7 Hz (log10 + 3) *P*/*P* ^FDR^	.262/.283	.072/.216	.283/.283
Alpha 8 to 12 Hz (log10 + 3) *P*/*P* ^FDR^	.034*/.051	.458/.458	.001**/.001**

*Note*: Statistics are derived from parametric one‐way repeated measures ANOVA's. *P* = *p* value, ^FDR^ = false discovery rate correction.

*p ≤ .05, **p ≤ .01, ***p ≤ .001.

### 3.5 Self‐report to the color condition

Currently the relationship between subjective emotional judgments and electrophysiological measures related to emotion is unclear. During the experiment participants self‐reported their emotional state using the Self‐Assessment Manikin. Self‐report of pleasure showed an effect of condition [*F*(2, 28) = 3.95, *p* = .037, *η* ^2^ = .189]. Post‐hoc comparisons revealed the only difference for self‐report of pleasure was an increase from resting state to control [*M*_diff_ = .722, *SE*_diff_ = .240, *t*(17.0) = 3.010, *p*_corrected_ = .024, 95% CI (.216, 1.23)]. We did not see any effect in self‐reported arousal [*F*(2, 34) = .791, *p* = .462, *η* ^2^ = .044] or dominance [*F*(2, 29) = 1.23, *p* = .300, *η* ^2^ = .068].

### 3.6 Summary

Overall, our findings show the blue condition in contrast to the white control increased autonomic response in skin conductance and respiration range. Although a decrease in mean values was found for heart rate variability, these were not significant. We also found the blue condition when compared to the control increased the EEG power spectral density in theta, alpha, and beta bandwidths, and increased left frontal hemispheric lateralization and frontal midline power in the alpha bandwidth. Lastly, we did not detect a relationship between self‐report of emotion between the blue condition to the control.

## 4 DISCUSSION

We investigated if there are detectable differences in emotion‐related autonomic and central nervous system processing when changing the design characteristic of color within a virtual built environment, and if participant changes in self‐reported emotion correspond with measures of autonomic and central nervous system modulations. We presented an interior built environment with minimal features to reduce contextual influence while controlling for comfort variables. Our findings showed that an autonomic measure of variability in heart rate (RMSSD) decreased when exposed to the blue condition, while measures of range in respiration and skin conductance response significantly increased. Power spectral density increased in the blue condition compared to the white across theta, alpha, and beta. As expected, we also found EEG frontal hemispheric lateralization and frontal midline power in the alpha bandwidth increased in the blue chromatic condition in contrast to the white achromatic control. Contrary to expectations, we did not detect evidence that color significantly affected SDRR, or skin conductance response and respiration mean values. The study also showed that participants' self‐report of emotion did not correspond with measures of autonomic response. This may reflect that we are unable to accurately identify and/or express our emotions to the built environment, or that the responses occur without conscious processing. Overall, this study demonstrates that color in the built environment modulates various indicators of neurophysiological activity in healthy adults.

The results of this study raise questions on how color exposure in the built environment may have a role in modulating our emotions. In emotional processing studies, higher frontal left hemispheric lateralization has been associated with positive emotions (Davidson, 1992; Ekman & Davidson, 1993). Given that stronger left lateralization was found in the blue condition, this might indicate the blue condition had a positive effect on participants. However, before claims can be made further work is needed to contrast the results with the effect of other hues, exposure time, type of activity being undertaken in the environment, and time of day. In contrast to the EEG results, increased range of skin conductance response and respiration have been shown to indicate elevated arousal and stress, which can have a negative impact on the body over long periods of time (Schneiderman et al., 2005). As we did not detect differences to the mean of autonomic measures, the significantly larger SCR and respiration range for the blue condition may simply reflect the perception of the changed environment rather than a distinct emotional response.

As mentioned, participants in this study were a subset of those who took part in our built environment scale study. Although the sample size for the color condition was smaller than that for the scale conditions, the color condition resulted in a clearer effect (Bower et al., 2021). While it is unclear why the effect is more pronounced, the division in dorsal and ventral visual streams involved in visual processing could account for the differences (Milner & Goodale, 2006). If this were the case, we might expect the ventral (perception) pathway to be primarily activated during color exposure (Kravitz et al., 2013), whereas the dorsal (action) pathway would be required during the processing of scale due to the need to determine distances and spaces in comparison to the body (Oliver & Thompson‐Schill, 2003). As it is suggested that the dorsal stream is associated with less visual awareness (Milner, 2012), and EEG is less likely to detect underlying brain activity, this could account for the difficulty in establishing autonomic differences to changing scales.

There have been different ways to approach study designs and reporting parameters when investigating the impact of color on emotional and neurophysiological responses. This may underpin the lack of consensus on the impact of built environment color at present. In this study, we selected a popular wall paint color for greater ecological validity, which we presented in the CAVE through projecting a LED image. Most studies previously conducted in virtual reality do not account for and explain how contextual features such as room function (clinical waiting area, school classroom, household bedroom, etc.) may influence responses, making it difficult to disentangle the role of built environment design from memories, associations, and preconceptions of the function and role of the built environment. This must be recognized as a critical study design parameter, as learnt autonomic response to a salient emotional association of the environmental context (i.e., fear of clinical waiting area) will confound responses measures (Baeuchl et al., 2015). We also raise concern where studies using virtual reality do not report the physical environment conditions (setting, people, and comfort variables) outside of the virtual space the study was conducted in, which also endangers ecological control.

Vast resources are consumed when creating our built environments. While these environments meet certain building codes and standards, there is no certainty in the impact they are having on our brain and body functioning. When altering or retrofitting a building, color is an economic and practical design characteristic to alter. For instance, changing the color of space through lighting or ink (paint, fabric, etc.) requires no changes to the structure and materiality of the building, whereas creating, for instance, a larger space with higher ceilings and curved panels may require floorplate demolition, specialized time‐intensive carpentry and will encounter constraints in the loadbearing structure. These variables also come with significant economic and embodied energy costs, adding to the structure's long‐term environmental sustainability. Changing built environment color also increases accessibility for a broader portion of the population to modify their environment, particularly those in the rental market and shared residences where there are limits to what can be altered. However, further studies are required to build complexity into experimental design to understand the interaction of design elements and social interactions before factoring in variables such as prior experiences (positive/negative conditioning from life experiences etc.).

This study has several limitations. Firstly, as the experiment also contained scale conditions, the blue color condition was presented at the end so we could not randomize the order of presentation. As a result, further work is required to rule out that the results are not due to a violation of expectation. However, as data were averaged across a two‐minute period it is less likely to have confounded the design compared to an event‐related potential study design. Next, as we selected a naturalistic design, we cannot disentangle the effects of hue, saturation, and brightness because the blue stimulus differed from the white stimulus in colorimetric dimensions. We also note that the room was not symmetrical in the furnishings present, with the built environment scene including a chair on the left‐hand side of the room. Although this could influence the regions of brain activity, our results indicated a leftward increase in EEG power spectra, which would not explain a visuospatial attention bias. Finally, this study used a sample of participants who identified as having ‘normal or corrected to normal vision’ and self‐reported they had no pre‐existing psychological, psychiatric, neurodevelopmental, and neurodegenerative conditions. Future studies should delve into larger inclusive samples, conduct an assessment to assess the participants’ color vision perception and ensure the colorimetric properties of the virtual environment are reported alongside the true colors from a spectrometer. This will enable us to break apart and understand the impact color has on a more representative sample of the population.

Although environmental enrichment studies explore whether environmental novelty is beneficial for cognitive function and mental health (Rojas‐Carvajal et al., 2022), it is still not well understood what the thresholds are between positive environmental stimulation effect and a negative stress response to the changes. Therefore, understanding what balance is needed for optimal mental health remains an unsolved yet important research question. While designers have long made claims about the emotional impact of the built environment on people, there has been a lack of empirical evidence investigating what impact design characteristics of the built environment have on emotional states and neurophysiological responses. While this study only investigated the impact of one hue, it provided the opportunity for us to gauge differences in the effect of color compared to scale on emotional and neurophysiological responses. This study provides evidence that the color of the built environment modulates biological systems associated with emotion and aids our understanding of the differences in processing different built environment design characteristics.

## AUTHOR CONTRIBUTIONS

**Isabella Simone Bower:** Conceptualization; data curation; formal analysis; funding acquisition; investigation; methodology; project administration; software; visualization; writing – original draft; writing – review and editing. **Gillian M Clark:** Formal analysis; resources; software; validation; writing – review and editing. **Richard Tucker:** Conceptualization; funding acquisition; investigation; supervision; writing – review and editing. **Aron T. Hill:** Formal analysis; resources; software; validation; writing – review and editing. **Jarrad Lum:** Formal analysis; resources; software; validation; writing – review and editing. **Michael A Mortimer:** Resources; software; writing – review and editing. **Peter G Enticott:** Conceptualization; data curation; formal analysis; funding acquisition; investigation; methodology; supervision; validation; writing – review and editing.

## FUNDING INFORMATION

I.S.B. is financially supported by a Deakin University Postgraduate Research Scholarship, Fellowship from the Academy of Neuroscience for Architecture (ANFA) and a Creativity and Innovation grant from Creative Futures Pty Ltd. P.G.E. was supported by a Future Fellowship from the Australian Research Council [FT160100077]

## Supporting information

Supinfo S1

## Data Availability

Source data and analysis code to accompany this manuscript submission are all available to be viewed on Open Science Framework: https://doi.org/10.17605/OSF.IO/FUDAC.
