# Supplementary material for: Built environment color modulates autonomic and EEG indices of emotional response
Source: Psychophysiology. 2022 Jun 20;59(12):e14121. doi: 10.1111/psyp.14121 (PMC9786701; doi:10.1111/psyp.14121)
Supplement: Supplementary file 1 — Supinfo S1 [file PSYP-59-e14121-s001.docx]

**Supplementary material**

**No significant correlation was found between time of day and autonomic response to blue room condition.**

To investigate whether there was a relationship between autonomic reactivity to the blue room condition and time of day the exposure occurred, we conducted an a-posteriori linear regression analysis. Using Pearson’s r correlations, we found no relationship between the time of day the testing was undertaken and participants autonomic response to the blue condition across measures of: HRV SDRR [r = -.040 p = .876, 95% CI (-.497, .435)], HRV RMSSD [r = -.225, p = .369, 95% CI (-.626, .270)], SCR mean [r = .270, p = .278, 95% CI (-.225, .655)], SCR mx-mn [r = -.243, p = .331, 95% CI (-.638, .253)], respiration mean [r = .182, p = .484, 95% CI (-.327, .610)], and respiration mx-mn [r = .158, p = .531, 95% CI (-.334, .582)].
